# Associations of loneliness with mental health and with social and physical activity among university students in Germany: results of the COVID-19 German student well-being study (C19 GSWS)

**DOI:** 10.3389/fpubh.2023.1284460

**Published:** 2023-11-10

**Authors:** Vanessa Wenig, Eileen Heumann, Christiane Stock, Heide Busse, Sarah Negash, Claudia R. Pischke, Katherina Heinrichs

**Affiliations:** ^1^Charité – Universitätsmedizin Berlin, corporate member of Freie Universität Berlin and Humboldt-Universität zu Berlin, Institute of Health and Nursing Science, Berlin, Germany; ^2^Unit for Health Promotion Research, University of Southern Denmark, Esbjerg, Denmark; ^3^Department Prevention and Evaluation, Leibniz Institute for Prevention Research and Epidemiology—BIPS, Bremen, Germany; ^4^Institute for Medical Epidemiology, Biometrics and Informatics, Interdisciplinary Center for Health Sciences, Medical School of the Martin-Luther University Halle-Wittenberg, Halle, Germany; ^5^Institute of Medical Sociology, Centre for Health and Society, Medical Faculty, Heinrich-Heine-University Duesseldorf, Duesseldorf, Germany

**Keywords:** loneliness, mental health, university students, COVID-19 pandemic, depressive symtoms, anxiety, physical activity

## Abstract

**Introduction:**

University students are at high risk for loneliness with a potential negative impact on health. The COVID-19 measures disrupted students’ academic routine and social life, which might have affected their perception of loneliness. This study investigated the prevalence of perceived loneliness among university students in Germany during the COVID-19 pandemic and its associations with mental health, behavioral outcomes, and sociodemographic characteristics.

**Methods:**

COVID-19 German student well-being study (C19 GSWS) collected data from five German universities from October 27th to November 14th, 2021, resulting in a sample of 7,203 respondents. Associations of loneliness with depressive symptoms, anxiety, social and physical activity, as well as sociodemographic characteristics, were analyzed using multivariable logistic regressions.

**Results:**

A total of 20.6% of students reported loneliness. Students with depressive or anxiety symptoms had more than eight- or sixfold odds, respectively, for reporting loneliness (depressive symptoms: OR = 8.29; 95% CI: 7.21–9.52; anxiety: OR = 6.48; 95% CI: 5.65–7.43) compared with students who did not report any symptoms. Students who were less physically active were more likely to experience loneliness compared with students who were more physically active (no moderate physical activity: OR = 1.39; 95% CI: 1.21–1.59; no vigorous physical activity: OR = 1.19; 95% CI: 1.04–1.36). We found no association between loneliness and social activity. However, loneliness was associated with being single (OR = 2.93; 95% CI: 2.55–3.36), living alone (OR = 1.31; 95% CI: 1.13–1.52), or having a temporary residency status in Germany (OR = 2.24; 95% CI: 1.65–3.04).

**Conclusion:**

Our findings highlight the importance of loneliness as a relevant factor associated with health. Further research is needed to determine potential protective factors to tackle loneliness and to investigate how study conditions at higher education institutions may affect students’ perceived loneliness.

## 1. Introduction

Feeling lonely is an unpleasant individual experience, which is not synonymous with social or objective isolation. Loneliness occurs when the network of social relations is quantitatively or qualitatively insufficient (1). Whether or not social networks are considered to be deficient depends on the individual relationship correlates (e.g., relationship aims, type of relationship) (2). Evidence suggests that a sense of belongingness or social connectedness might act as a buffer against loneliness (3). Conversely, the lack of social connectedness might result in feelings of loneliness (4).

Evidence further suggests that loneliness is associated with an increased all-cause mortality (5), being female (6–9), living alone (7–9), and being single (8, 9). Loneliness is also strongly linked to mental health: depression, generalized anxiety disorder, and suicidality have been shown to be strongly associated with loneliness (8, 10, 11). Moreover, loneliness is an important predictor of long-term health and is not only limited to older individuals (12). Adolescents and young adults are also widely affected by feelings of loneliness, and strong associations with depressive symptoms and anxiety have been demonstrated (12, 13). Generally, previous research indicated that loneliness, anxiety, and depression were distinct but interrelated phenomena (11, 14). Furthermore, loneliness in youth is a relevant predictor of the health status in adulthood (15) and correlates with future mental health problems. As the duration of loneliness in youth seems to be an important predictor for depression later in life, the prevention of loneliness among young people is a pressing issue (16, 17). It appears, therefore, important to identify and address loneliness at an early stage in childhood or in young adulthood in order to prevent its negative effects on mental health later on in life.

The prevalence of perceived loneliness and mental illness among young adults, in particular among university students, is generally at high levels (18–20). Former studies on loneliness among university students and young adults examined associations with age, gender, living situation, relationship status, immigration status, and mental health problems. In general, younger age groups were found to be more likely to experience loneliness (6, 7, 9, 21). Within this age group, younger and older students were reported to have higher feelings of loneliness compared with middle-aged students (19), indicating a U-shaped association between age and loneliness among university students. Similarly, being female (22, 23), living alone (18, 19, 24), being single (18, 19, 23, 25), and studying abroad (19) were associated with more feelings of loneliness. In contrast, some studies could not confirm these associations between loneliness and gender among higher education students (18, 25).

Loneliness is a mental health issue that has received particular attention during the COVID-19 pandemic (26). The pandemic caused governments to implement measures to contain the disease such as school and university closures and social distancing. In Germany, the first lockdown started in March 2020 with easing steps over the subsequent summer. When the incidence rates increased again in autumn 2020 (27), the second lockdown began and lasted until May 2021. Whereas elementary and secondary school students had already been able to return to their institutions earlier, higher education institutions remained closed and, thus, online teaching continued. From April 2022 onwards, universities were reopened throughout Germany and returned to face-to-face teaching.

Some studies found that during the pandemic, social isolation and its consequences led to increased prevalence rates of loneliness (28–30). Especially for children and adolescents, the disease containment measures had effects on their mental health and were associated with increased loneliness (16, 29, 30). Even before the pandemic, loneliness was shown to pose significant health risks in terms of anxiety and depressive symptoms for young adults and students (12, 16, 18). During the pandemic, an increase of mental health issues (31) and loneliness (31, 32) among university students was observed. The pre-existing predictors of loneliness observed prior to the pandemic appeared to remain unchanged throughout the course of the pandemic: Bu et al. (33) found that being female or of younger age, living alone, having lower education or income, and belonging to ethnic minorities were risk factors for loneliness.

To reduce or prevent loneliness, recent studies revealed the benefits of social networks regarding life satisfaction and well-being (34, 35). The social interactions during physical activity (e.g., with other participants or with an instructor) could influence individuals’ perceptions of social support (36). In this sense, physical activity could offer various relationship opportunities and could create a sense of belonging (37). The evidence of the association between physical activity and loneliness is inconclusive. On the one hand, physical activity might reduce feelings of loneliness; on the other hand, loneliness may decrease the engagement in physical activity (38). In university students, physical activity seems to be a protective factor against loneliness (18, 39). A low level of physical activity (less than 1 hour per week) was associated with loneliness (18). However, Jennen et al. (40) found that just being physically active was insufficient to have an effect on loneliness. Another study found that young adults had to experience physical activity as enjoyable in order to experience decreased feelings of loneliness (41).

The literature regarding the impact of social contact on loneliness among university students is mixed. Generally, work by Diehl and Hilger (24) revealed that the transitional phase between school and university is often connected with a change of residency and, thus, the loss of existing social networks and close family connections. In a Finnish study, loneliness was associated with less social contact with friends in younger ages (21). However, especially for students, friendships and frequent social contact were beneficial to their mental health during the pandemic (42). Rumas et al. (43) found that a larger social network was accompanied by less loneliness, but frequent virtual contact did not help to reduce loneliness. Earlier studies found that the lack of quality of social contact, rather than quantity, was associated with loneliness (41).

Overall, we conclude from the present literature that university students are at risk of exposure to loneliness and its negative health outcomes. Beutel et al. (8) noted that loneliness should be regarded as a relevant health variable on its own. In order to address the research gaps regarding loneliness and mental health, and the role of social networks, and physical activity for loneliness among university students, the aims of this study were (1) to examine the prevalence of perceived loneliness among German university students in a later phase of the COVID-19-pandemic and (2) to identify factors associated with loneliness. Factors of interest included (2a) anxiety and depressive symptoms, (2b) social and physical activities as well as (2c) sociodemographic characteristics. First, we expected anxiety and depressive symptoms to be positively associated with loneliness. Second, we hypothesized that students who engaged in at least one social activity per week are less likely to feel lonely. In addition, our third hypothesis was that students who were physically active were also less likely to experience feelings of loneliness.

## 2. Materials and methods

### 2.1. Study design and procedures

The COVID-19 German student well-being study (C19 GSWS) is a cross-sectional study and followed the COVID-19 International Student Well-being Study (C19 ISWS) (44). The online questionnaire of the C19 GSWS was implemented at five German universities: Charité – Universitätsmedizin Berlin, University of Bremen, University of Siegen, Martin-Luther-University Halle/Wittenberg, and Heinrich-Heine-University Düsseldorf. Using LimeSurvey, data collection was conducted at the same time at all five participating universities between October 27th and November 14th, 2021, i.e., at the beginning of the winter semester. During this time, the learning and teaching situation at German universities varied widely due to different regional COVID-19 regulations. In general, face-to-face interaction was limited in favor of online teaching: only few seminars with smaller learning groups were offered in person, whereas most of the lectures were held remotely throughout the whole winter semester.

The questionnaire used was a modified version of the C19 ISWS questionnaire. The core questionnaire used can be found elsewhere (45). The participants invited were students aged 18 years and above who were enrolled at one of the five universities. University students were invited to participate in the online survey via e-mail, e-learning platforms (Martin-Luther-University Halle/Wittenberg and University of Bremen), or via Instagram (Heinrich-Heine-University Duesseldorf). Students had the option of answering the survey in German or English. More information about the design and recruitment of the C19 GSWS study is available elsewhere (46). Further, the dataset is openly accessible via 10.5281/zenodo.7659845 (47).

All participants gave their informed consent before participating in the survey. The ethics committees of the five participating universities have obtained ethical approval (University of Bremen 2021-28-EIL, University Halle-Wittenberg 2020–066, Heinrich-Heine-University Duesseldorf 2020-958_1, Charité – Universitätsmedizin Berlin and University of Siegen have accepted the ethic vote of the University of Bremen). We used the Strengthening the Reporting of Observational Studies in Epidemiology (STROBE) guideline for reporting this cross-sectional study (48).

### 2.2. Measures

#### 2.2.1. Loneliness

Loneliness was assessed with a single item from the Center for Epidemiologic Studies Depression Scale (CES-D) (49): ‘Please indicate how much of the time during the past week you felt lonely’. Response options included: ‘none or almost none of the time’, ‘some of the time’, ‘most of the time’, and ‘all or almost all of the time’. Responses were converted to binary coding to allow for a comparison of those who reported feeling lonely most, almost all and all of the time (in the following referred to as ‘major loneliness’) with those who reported feeling lonely ‘none or almost none of the time’ or ‘some of the time’ (in the following referred to as ‘minor loneliness’ (reference category)).

#### 2.2.2. Anxiety and depressive symptoms

Anxiety symptoms were assessed with the 2-item Generalized Anxiety Disorder Scale (GAD-2), which is based on the GAD-7 (50). The GAD-2 is a valid and reliable instrument for assessing generalized anxiety symptoms in a university context (51). The GAD-2 was conducted with the following basic question: ‘Over the last 2 weeks, how often have you been bothered by the following problems’ and the two items were ‘feeling nervous, anxious, or on edge’ and ‘not being able to stop or control worrying’. For each item, there were the following answer options: (0) ‘not at all’, (1) ‘several days’, (2) ‘more than half the days’, and (3) ‘nearly every day’. The GAD-2 sum score can range from 0 to 6, and as suggested in the literature, we chose a cut-off point of 3 (50) to indicate whether the participants showed anxiety symptoms (0 to 3 ‘no anxiety symptoms’ (reference category); 4 to 6 ‘anxiety symptoms’). The Cronbach’s alpha in our sample was 0.85 for GAD-2.

Depressive symptoms were measured with the short-form version of the Patient Health Questionnaire (PHQ-2) (50, 52). The PHQ-2 includes the first two items (‘feeling down, depressed, or hopeless’ and ‘little interest or pleasure in things’) of the PHQ-9, and we used the same basic question, response options, and cut off as for GAD-2 (53). In addition, the PHQ-2 is also validated in the university context (54). The Cronbach’s alpha in our sample was 0.79 for PHQ-2.

#### 2.2.3. Social activity

A new variable was generated based on 10 items assessing social activity. Participants were asked to indicate whether, in the last week, they had engaged in any of the following activities: (1) a walk with another person; (2) a bike ride with another person; (3) drinks or a picnic with friends or family; (4) talked to friends or family on the street; (5) participated in a recreational class online (e.g., yoga, aerobics, fitness); (6) played a game or a quiz online with friends or family; (7) talked to friends or family through a video-call; (8) talked to friends or family over the phone; (9) chatted with friends or family online (excluding video-calls or phone calls); (10) none of the above. Multiple responses were possible. First, we summed all social activities per participant and second, chose a cutoff >0, similar to Nyqvist et al. (21). This resulted in a new variable with two categories: those who had participated in at least one social activity (reference category) and those who had not participated in any activity in the previous week.

#### 2.2.4. Physical activity

In our study, physical activity was assessed using two items: ‘On average, during the last week, how often did you perform vigorous physical activities like lifting heavy things, running, aerobics, or fast cycling for at least 30 min?’ and ‘On average, during the last week, how often did you perform moderate physical activities like easy cycling or walking for at least 30 min?’. For each item, there were the following answer options: (1) (almost) never; (2) less than once a week; (3) once a week, (4) more than once a week; (5) (almost) daily. For the analysis, we recoded the variables into a binary variable. As suggested by Shankar et al. (55), participants who reported moderate or vigorous physical activity only once a week or less (answers 1–3) were classified as not meeting the criteria for being physically active. Participants who reported levels of physical activity (answers 4–5) were classified as being physically active (reference category).

#### 2.2.5. Sociodemographic characteristics

We included the following variables in our analyses: self-identification with gender (‘female’, ‘male’, ‘diverse’), age (categorized into ‘between 18 and 20 years old’, ‘between 21 and 25 years old’, and ‘aged 26 and older’, as done by Hysing et al. (19)), relationship status (‘single’, ‘in a relationship’, ‘it is complicated’), residence status in Germany (‘permanent residency’ and ‘temporary residency’), and living situation (‘living alone’ and ‘living with other persons in the household’).

### 2.3. Data analyses

First, frequencies were calculated for sociodemographic characteristics, as well as prevalence of loneliness, by the different sociodemographic characteristics. Second, a multivariable logistic regression model was employed to examine the associations of social activity, physical activity, and sociodemographic variables as independent variables with loneliness as dependent variable. The co-variates included in the model were age, gender, relationship status, living situation, and residency status. Thus, the regression model was adjusted for all variables included simultaneously in a single block. Third, two multivariable logistic regression models for anxiety and depressive symptoms as independent variables were carried out to determine associations with loneliness as dependent variable, adjusting for sociodemographic characteristics (age, gender, relationship status, living situation, and residency status). Respondents with missing values in the variables of interest were excluded from the regression models. Before entering the independent variables into the models, we tested for multicollinearity. Correlations between the independent variables were low (*r* < 0.70), indicating that multicollinearity was not a confounding factor in the analysis. The results from the logistic regression analyses were presented as odds ratios (ORs) with 95 percent confidence intervals (CIs). All statistical analyses were performed with IBM SPSS, version 26, on a Windows 10 Education system.

## 3. Results

The sociodemographic characteristics of the sample are presented in Table 1. Of the 7,203 students in the sample, most identified themselves as female (67.9%) and were between 21 and 25 years old (54.4%). A little more than half of the participants were not in a steady relationship and were either single (52.8%) or had a complicated relationship status (4.2%). Further, most of the participants lived together with others (78.8%) and had a permanent residency in Germany (96.3%). Almost half of the students were currently enrolled in a bachelor’s program (47.2%), one quarter was enrolled in a health-related field of study (26.4%), and the largest proportions were studying in Halle/Wittenberg (30.1%), Bremen (25.3%) and Siegen (21.7%). Most of the students (91.0%) participated in at least one social activity within the last week. Regarding moderate and vigorous physical activity in the last week, 31.7% or 58.1%, respectively, of the participants were physically inactive.

**Table 1:** Characteristics of the sample (*n* = 7,203).

Variables	*n*	%
Gender (*n* = 7,100)		
Male	2,199	31.0
Female	4,824	67.9
Diverse	77	1.1
Age (*n* = 7,181)		
18–20	1,434	20.0
21–25	3,906	54.4
≥ 26	1,841	25.6
Relationship status* (*n* = 7,062)		
In a steady relationship	2,963	41.2
Single	3,797	52.8
It is complicated	302	4.2
Living situation (*n* = 6,992)		
Alone	1,482	21.2
With others	5,510	78.8
Residency status in Germany* (*n* = 7,165)		
Permanent residency	6,927	96.3
Temporary residency	238	3.4
Degree program (*n* = 6,996)		
Bachelor	3,305	47.2
Master	1,385	19.8
State exam	2,306	33.0
Study field (*n* = 7,203)		
Health-related	1,905	26.4
Other	5,298	73.6
University (*n* = 7,203)		
University of Bremen	1,819	25.3
Charité – Universitätsmedizin Berlin	1,131	15.7
Heinrich-Heine-University Düsseldorf	520	7.2
Martin-Luther-University Halle/Wittenberg	2,168	30.1
University of Siegen	1,565	21.7
Social activity (*n* = 6,975)		
No social activities last week	626	9.0
1 or more social activities last week	6,349	91.0
Moderate physical activity (*n* = 7,163)		
Physically inactive	2,274	31.7
Physically active	4,889	68.3
Vigorous physical activity (*n* = 7,127)		
Physically inactive	4,141	58.1
Physically active	2,986	41.9

*Missing percentages are due to answer options ‘no information’ or ‘I do not know’.

Table 2 presents self-reported major loneliness in the overall sample. In total, 20.6% of the students reported major loneliness in the past week. Feelings of major loneliness were more prevalent among participants being single (29.6%), living alone (26.2%), or having a temporary residency in Germany (38.1%). See prevalence of major loneliness by sociodemographic characteristics in Table 3.

**Table 2:** Prevalence of loneliness in the sample (*n* = 6,928).

Loneliness during the past week in the sample	*n*	%
Minor loneliness (Defined as: none or almost none of the time, some of the time)	5,504	79.4
Major loneliness (Defined as: most, all or almost all of the time)	1,424	20.6

**Table 3:** Prevalence of major loneliness by sociodemographic characteristics.

Major loneliness by sociodemographic characteristics	*n*	%*
Gender (*n* = 1,400)		
Male	439	20.8
Female	940	20.2
Diverse	21	28.0
Age (*n* = 1,418)		
18–20	311	22.5
21–25	766	20.4
≥ 26	341	19.3
Relationship status (*n* = 1,396)		
In a steady relationship	447	12.2
Single	844	29.6
It is complicated	105	20.6
Living situation (*n* = 1,393)		
Alone	376	26.2
With others	1,017	19.2
Residency status in Germany (*n* = 1,410)		
Permanent residency	1,324	19.9
Temporary residency	86	38.1
Degree program (*n* = 1,390)		
Bachelor	725	22.9
Master	253	19.1
State exam	412	18.4

*Percentage within the variable gender, age, relationship status etc.

Table 4 presents the results of the multivariable logistic regression analysis to determine the associations of social activity, physical activity, and sociodemographic characteristics with loneliness as dependent variable. There was no association between feelings of loneliness and participation in social activities. Being physically inactive was associated with major loneliness (no moderate physical activity: OR = 1.39; 95% CI: 1.21–1.59; no vigorous physical activity: OR = 1.19; 95% CI: 1.04–1.36). Furthermore, the analysis showed that the odds of experiencing major loneliness increased for students being single (OR = 2.93; 95% CI: 2.55–3.36), reporting a complicated relationship status (OR = 3.86; 95% CI: 2.94–5.08), living alone (OR = 1.31; 95% CI: 1.13–1.52), or having a temporary residency in Germany (OR = 2.24; 95% CI: 1.65–3.04).

**Table 4:** Associations between social activity, moderate and vigorous physical activity and loneliness as dependent variable: a multivariable logistic regression model (*n* = 6,396).

Variables		Loneliness	
		OR*	95% CI
Social activity	1 or more social activities last week (ref.)	1.00	
	No social activities last week	1.07	(0.86–1.34)
Moderate physical activity	Physically active (ref.)	1.00	
	Physically inactive	1.39	(1.21–1.59)
Vigorous physical activity	Physically active (ref.)	1.00	
	Physically inactive	1.19	(1.04–1.36)
Age	18–20 (ref.)	1.00	
	21–25	1.01	(0.86–1.20)
	≥ 26	1.03	(0.85–1.25)
Gender	Male (ref.)	1.00	
	Female	1.10	(0.95–1.26)
	Diverse	1.42	(0.80–2.53)
Relationship status	In a steady relationship (ref.)	1.00	
	Single	2.93	(2.55–3.36)
	It is complicated	3.86	(2.94–5.08)
Living situation	With others (ref.)	1.00	
	Alone	1.31	(1.13–1.52)
Residency status in Germany	Permanent residency (ref.)	1.00	
	Temporary residency	2.24	(1.65–3.04)

OR, odds ratios; CI, confidence interval; ref., reference category; *ORs adjusted for all other variables in the table.

Table 5 and Table 6 present the results of the regression models analyzing the associations between depressive symptoms and anxiety, respectively, as independent variables, and loneliness as dependent variable, while controlling for sociodemographic variables. We found a more than eightfold chance of suffering from loneliness among students who reported depressive symptoms (OR = 8.29; CI: 7.21–9.52), compared with students in the reference group. Reporting anxiety symptoms was also associated with a more than sixfold likelihood for reporting loneliness (OR = 6.48; CI: 5.65–7.43).

**Table 5:** Associations between depressive symptoms and loneliness as dependent variable: a multivariable logistic regression model (*n* = 6,499).

Variables		Loneliness	
		OR**	95% CI
Depressive symptoms (PHQ-2*)	No depressive symptoms (ref.)	1.00	
	Depressive symptoms	8.29	(7.21–9.52)
Age	18–20 (ref.)	1.00	
	21–25	0.95	(0.80–1.14)
	≥ 26	0.97	(0.79–1.20)
Gender	Male (ref.)	1.00	
	Female	1.02	(0.88–1.19)
	Diverse	0.91	(0.49–1.70)
Relationship status	In a steady relationship (ref.)	1.00	
	Single	3.24	(2.80–3.76)
	It is complicated	3.49	(2.58–4.71)
Living situation	With others (ref.)	1.00	
	Alone	1.36	(1.16–1.59)
Residency status in Germany	Permanent residency (ref.)	1.00	
	Temporary residency	2.15	(1.54–3.01)

OR, odds ratios; CI, confidence interval; ref., reference category; *cut off point of 3; **ORs adjusted for all other variables in the table.

**Table 6:** Associations between anxiety and loneliness as dependent variable: a multivariable logistic regression model (*n* = 6,498).

Variables		Loneliness	
		OR**	95% CI
Anxiety (GAD-2*)	No anxiety (ref.)	1.00	
	Anxiety	6.48	(5.65–7.43)
Age	18–20 (ref.)	1.00	
	21–25	1.00	(0.85–1.19)
	≥ 26	1.00	(0.81–1.23)
Gender	Male (ref.)	1.00	
	Female	0.89	(0.77–1.04)
	Diverse	0.70	(0.39–1.26)
Relationship status	In a steady relationship (ref.)	1.00	
	Single	3.14	(2.71–3.63)
	It is complicated	3.64	(2.71–4.89)
Living situation	With others (ref.)	1.00	
	Alone	1.37	(1.18–1.61)
Residency status in Germany	Permanent residency (ref.)	1.00	
	Temporary residency	2.21	(1.60–3.01)

OR, odds ratios; CI, confidence interval; ref., reference category; *cut off point of 3; **ORs adjusted for all other variables in the table.

## 4. Discussion

This study investigated the prevalence of loneliness among university students and its association with mental health, social and physical activity, as well as sociodemographic characteristics, during the late phase of the COVID-19 pandemic at five German universities using the C19 GSWS dataset.

With respect to our first study objective, we found that one-fifth of the respondents reported feelings of loneliness most or almost all the time. Our findings are consistent with and add to previous work showing that the prevalence of loneliness among students is similarly high as before the pandemic in, e.g., Norway (19) and Iran (56). Some previous research showed a lower pre-pandemic prevalence of loneliness among students in Germany (18) and another study, investigating the prevalence of loneliness in the adult population, showed that it was only half that reported in our study (8). It is difficult, however, to make direct comparisons, because different studies used various ways to measure the prevalence of loneliness. In addition, it is important to consider, however, that the risk of infection with the potentially lethal coronavirus caused anxiety and self-isolation (57). Therefore, social isolation can be considered as a normal, non-pathological reaction to cope with the risk of infection during the pandemic (9). According to Shiovitz-Ezra and Ayalon (58), situational loneliness is a temporary experience due to a major change in social life but with the likelihood of fast remission. While situational loneliness might not be a severe problem, suffering from feelings of loneliness over a long period of time could lead to chronic loneliness. Finally, chronic loneliness increases the overall mortality risk (58) and is associated with future mental health problems (16). It remains unclear from our data whether the loneliness reported by our study participants reflects situational or chronic loneliness. As the studies of Zahedi et al. (56) and Hysing et al. (19) showed similar prevalence rates of loneliness among university students before the pandemic, we assume that both situational and chronic loneliness may have contributed to the prevalence rate observed.

Regarding our first hypothesis, our findings are in agreement with the hypothesis and with previous research showing that loneliness is associated with depressive symptoms and anxiety (12, 13). Previous research reported loneliness, anxiety, and depression to be interrelated (11, 14). However, similar to Lee et al. (29), our results also suggest that loneliness could be a crucial mechanism for the increase in depressive symptoms during the pandemic. An important consideration in interpreting the results is that depression is likely to make people rate their social support as insufficient, to let them withdraw from their social network, and to make them feel lonely (10). It is possible that students with stronger social networks experienced greater disruption in their social lives and, as a result, felt lonelier during the pandemic (29). In this context, previous research emphasized that especially COVID-19-specific worries, social and physical isolation, and the lack of interaction were associated with negative mental health outcomes for students (23).

A second aim (2b) of our study was to examine associations between loneliness and social and physical activity. In contrast to our second hypothesis, our analyses did not reveal an association between social activities and loneliness. However, we were only able to consider the number of weekly activities in our analyses. Previous research suggests that simply increasing the number of social contacts is unlikely to be sufficient to reduce loneliness, because loneliness can also be experienced in the company of other people (12). Further, previous research indicated that the quality of social contact, rather than the quantity, is a predictor of loneliness (41, 59). However, the results of Elmer et al. (23) suggested that students with smaller personal networks were more likely to become lonely during the pandemic. In addition, during the pandemic, students were forced to use digital communication with their social networks, and they may have experienced this shift in communication and social interaction as both negative and positive (60). Studies on older adults showed that sharing activities and experiences with peers created a sense of belonging and could decrease feelings of loneliness during the pandemic (61). In addition, Masi et al. (62) found in their meta-analyses, among others, that interventions increasing opportunities for social interaction could reduce loneliness for different age groups. However, the literature on loneliness interventions is inconsistent and mainly available for older age groups (63).

With respect to physical activity, however, we found support for our third hypothesis that loneliness and physical activity were inversely related. Our results are in line with previous research showing that students’ physical activity seems to protect against loneliness (18, 39). Prior evidence showed that the way students experience physical activity is also very important to decrease feelings of loneliness (40). Students may compensate any lack of trustworthy friendships and meaningful social interactions with social bonds in team sports (39). Previous studies suggested that students’ physical activity decreased during the pandemic (64). It is possible that team athletes, in particular, experienced greater social isolation and loneliness during the pandemic, because COVID-19 measures included social distancing and cancelation of team sport activities. More research is needed to disentangle the interplay between different types of physical activity and loneliness.

Regarding sociodemographic variables and their association with loneliness, our results are in line with previous research showing that students being single (18, 19, 23, 25), living alone (18, 19, 24), and studying abroad, i.e., having a temporary residence status (19), are more likely to suffer from loneliness. Moreover, we found no association between gender or age and loneliness, which is consistent with some previous studies (18, 25). Other studies, however, describe that students who are female or younger are more affected by loneliness (22, 23). Such associations with gender and age may be attributed to different sample compositions, a gender imbalance in the samples, and time of data collection. Labrague et al. (22) studied a sample of nursing school students with a high percentage of female students, and Elmer et al. (23) studied a sample of students, mainly from engineering and science programs with a low percentage of female students. Age and gender may be relevant determinants of loneliness within different subject groups. Further, both studies were conducted during the first lockdown in April 2020 (22, 23). This was the students’ first exposure to closed campuses and online teaching. Before the COVID-19 pandemic (18) and later during the first lockdown (23), gender and age were not associated with loneliness. At the time of our survey, it was the fourth semester under the COVID-19 restrictions, so younger and female students may have been able to develop better coping strategies to deal with social isolation.

Overall, our findings confirm that loneliness is a severe mental health outcome among university students, and early intervention is needed to prevent loneliness from persisting over an extended period of time. Our results suggest that close social relationships seem to be an important protective factor, while the number of social activities does not appear to play a significant role. During the pandemic, when students were forced to follow social distancing measures, it seemed to make a considerable difference whether they lived alone, were single, or were international students. Health promotion programs should focus on the role of friendship and promote social contact, especially during the transition phase from school to university and particularly target international students. Overall, physical and social activity may help to connect students in the setting of their university and can be addressed in student health programs. Interventions to reduce loneliness should focus on improving social skills and increasing social support and opportunities for social contact including group based physical activity (62).

## 5. Strengths and limitations

The multi-center COVID-19 German student well-being study (C19 GSWS) contributes to the existing knowledge on associations of loneliness with depressive symptoms, anxiety and physical activity among university students in Germany during the pandemic based on a large sample. Despite these strengths, the current results could not analyze any differences according to teaching situations across universities during the pandemic and should be interpreted with consideration of several limitations. First, we cannot make a causal claim due to the cross-sectional design. Longitudinal research is needed to distinguish between situational and chronic loneliness, as chronic loneliness has a major impact on health outcomes later on in life. Second, this study used a single-item measure of loneliness, a question from the CES-D (49), which might explain the strong associations of loneliness with mental health outcomes. However, using one single item measure for loneliness is common (8, 58), including the university context (19, 65). Future studies could validate or compare the single item measurement to other validated measures such as the University of California, Los Angeles Loneliness Scale (UCLA Loneliness Scale) (66). Third, the C19 GSWS was performed with a convenience sample and, thus, a selection bias cannot be ruled out. This might have affected the prevalence rates reported in this study. Students with severe loneliness could be less likely to participate which would have led to an under-reporting of loneliness. However, we assume that the effect of any selection bias on the reported associations is low. More than a quarter of the participants were university students of medicine or health-related subjects. Hence, the results are not representative of the general German student population. Similarly, our sample had a higher proportion of female participants which resulted in a gender imbalance. Previous studies have shown the same gender distribution: women are more likely to participate in health-related research (67). However, the effects of this imbalance on the associations presented can be considered as low, since the analysis was adjusted for gender and no significant gender differences in loneliness were found. Furthermore, the present study used self-reported measures. The PHQ-2 and GAD-2 measured the symptoms of the last 2 weeks; the items assessing loneliness, physical activity and social activity only referred to the last week. Although the PHQ-2 and GAD-2 are well-validated scales, interview-based scales are the gold standard for mental health assessment.

## 6. Conclusion

The present study underlines the importance of loneliness as a relevant health variable among university students. About one in five students reported major feelings of loneliness during the pandemic. We found associations of loneliness with depressive symptoms, anxiety, and physical activity. Loneliness among university students was linked to being single or having a complicated relationship status, living alone, or having a temporary residency in Germany. Unlike other previous research, we did not find associations between loneliness and participation in social activities. Moreover, our results could not identify gender and age as correlates of loneliness among university students. Further research is needed to study potentially protective factors and to investigate how conditions at universities may affect loneliness among students. Students’ health management programs should implement interventions to tackle loneliness and to build a health-promoting study environment.

## Data availability statement

The dataset presented and analyzed in this study can be found in an online repository, namely Zenodo: https://zenodo.org/record/7659846.

## Ethics statement

The studies involving humans were approved by University of Bremen 2021-28-EIL, University Halle-Wittenberg 2020-066, Heinrich-Heine-University Duesseldorf 2020-958_1, Charité – Universitätsmedizin Berlin and University of Siegen have accepted the ethic vote of the University of Bremen. The studies were conducted in accordance with the local legislation and institutional requirements. The participants provided their written informed consent to participate in this study.

## Author contributions

VW: Conceptualization, Methodology, Visualization, Writing – original draft. EH: Conceptualization, Data curation, Writing – review & editing. CS: Conceptualization, Writing – review & editing. HB: Conceptualization, Writing – review & editing. SN: Conceptualization, Writing – review & editing. CP: Conceptualization, Writing – review & editing. KH: Conceptualization, Methodology, Supervision, Writing – review & editing.
